# Transcatheter Coil Embolization of Single Coronary Artery Fistula Using the Occlusion Test

**DOI:** 10.1155/2018/7505283

**Published:** 2018-05-22

**Authors:** Shin Takahashi, Yurie Takizawa, Satoshi Nakano, Junichi Koizumi, Kotaro Oyama

**Affiliations:** ^1^Department of Pediatrics, School of Medicine, Iwate Medical University, Morioka, Japan; ^2^Department of Cardiovascular Surgery, School of Medicine, Iwate Medical University, Morioka, Japan

## Abstract

The case of a patient in whom hemodynamic and electrocardiographic studies using the occlusion test for coronary artery fistulas (CAF) were safely performed prior to catheter embolization is reported. A 1-year-old girl had a separate right coronary artery arising from a left single coronary artery that formed a significant coronary artery fistula to the right ventricle. Coronary steal by the large coronary artery fistula narrowed the left coronary artery. The right coronary artery branches could not be clearly identified due to an overlap with the fistula. Due to the long porous CAF, embolic procedures could cause serious complications. We confirmed the safety by performing an occlusion test of the CAF's proximal blood vessels. Following total occlusion of the CAF for 10 minutes, pulmonary arterial pressure and aortic blood pressure were not significantly changed. No bradycardia, atrioventricular block, or ST changes were observed. Coil embolization treatment was performed safely. For patients with long distal CAF complicated with a single coronary artery, myocardial ischemia and conduction system disorders can be identified by performing the occlusion test before embolization.

## 1. Introduction

Coronary artery fistula (CAF) has congenital and acquired coronary artery abnormalities. This abnormality accounts for 0.27–0.4% of all congenital cardiac defects. Congenital single coronary artery comprises about 0.04% of congenital cardiac anomalies [1]. In addition, CAF complicated with single coronary artery is rare. Myocardial ischemia due to coronary steal is an extremely difficult problem, and its presence can be an indication for treatment. Acute complications associated with embolization of CAF include myocardial ischemia and conduction system disorders [2]. A case of significant CAF is presented in a heart with normal structure. A hemodynamic study involving an occlusion test of the fistula's proximal vessel prior to catheter embolization was performed, and the patient safely underwent catheter treatment.

## 2. Case Presentation

The parents of the following patient have given their consent for the publication of this report.

### 2.1. Case

**Figure 1:**
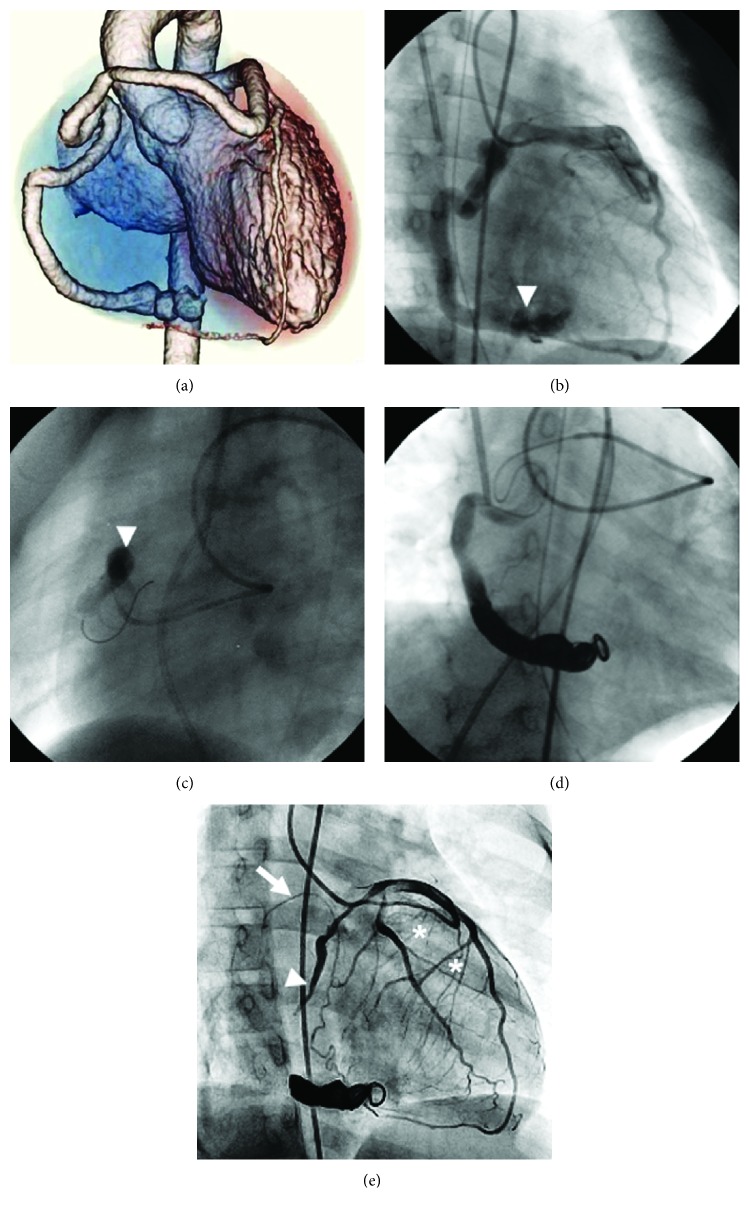
(a) 320-row coronary CTA. The single coronary artery view shows the anterior course of the separate right coronary artery coming off the LAD. This is the anterior view. (b) Selective coronary angiography of the LMT shows the LAD and the LCX with weak contrast effects and a CAF with “multiple caliber change” of the RCA to the right ventricle (arrowhead). (c) Occlusion test on the proximal site of CAF using an occlusion balloon (arrowhead). (d) Engaged microcatheter in the distal RCA and embolization using a detachable coil. (e) One year after coil embolization, selective LMT coronary angiography clearly distinguishes the contrast effect of the LAD and LCX, the sinus node branch branching from LCX (white arrow), and right ventricular branches from the proximal side of the RCA (asterisks). The proximal end of the coil-embolized CAF formed a thrombus and occluded (arrowhead). CAF: coronary artery fistula; CTA: computed tomography angiography; LAD: left anterior descending coronary artery; LCX: left circumflex coronary artery; LMT: left main coronary trunk artery; RCA: right coronary artery.

A 1-year-old girl presented with continuous heart murmur. Chest radiography showed slight cardiomegaly (cardiothoracic ratio, 55%). Electrocardiogram showed sinus rhythm and no ST changes. Echocardiography showed a dilated left main coronary trunk artery (LMT) and a right coronary artery (RCA) entering a right ventricular fistula. Coronary computed tomography angiography (CTA) with three-dimensional volume rendering revealed the thick and torsional RCA originating from the left anterior descending coronary artery (LAD), with fistulous communication to the right ventricle via a large vessel (Figure 1(a)). In cardiac catheterization, the Qp/Qs was 2.6. The CAF was a “beaded and caliber change” form. Coronary angiography could not distinguish the RCA's peripheral branches (Figure 1(b)). The sinus node branch, right ventricular branches, and acute marginal branch from the RCA could not be clearly identified. Additionally, neither the atrioventricular nor the posterior descending branch on the peripheral site could be identified due to an overlap with the fistula. Collateral vessels from the LCA to the same site also could not be identified. Because of the long porous CAF, there was a concern about the embolism disrupting peripheral branch blood flow. We tried the occlusion test on the proximal vessel of the CAF. Using the PercuSurge GuardWire™ system (Medtronic, Santa Rosa, CA, USA), an occlusion test was performed in the proximal site of the RCA using a 6 mm balloon (Figure 1(c)). Following total occlusion for 10 minutes, the pulmonary arterial pressure (systolic/diastolic/mean) changed from 19/10/15 to 18/10/14 mmHg and the aortic blood pressure changed from 109/49/64 to 109/57/66 mmHg, both of which had no significant changes. In addition, no bradycardia, atrioventricular block, or ST changes were observed on electrocardiogram. Coil embolization was performed with GDC™ Detachable Coils (Boston Scientific, Fremont, CA, USA) (Figure 1(d)). Because of the long porous CAF, the target vessel was an embolus of a long lumen. However, the embolic area did not cross the balloon occlusion site. Coil embolization treatment techniques were safely performed.

After this procedure, anticoagulation therapy was continued. In cardiac catheter examination one year after coil embolization, the contrast effect of the LAD and left circumflex coronary artery (LCX) increased, and each coronary branch was easy to distinguish. The residual shunt from the CAF did not have a contrast effect. The proximal end of the coil-embolized CAF formed a thrombus and occluded. However, there existed right ventricular branches from the proximal side of the RCA. The sinus node branch revealed branching from the LCX. The collateral vessels from the developed septal branch, LAD, and sinus node branch supplemented the peripheral areas of the RCA (Figure 1(e)). The patient's hemodynamics are now stable, and she is in good health.

## 3. Discussion

Up to 57% of patients with a CAF also have another congenital cardiovascular anomaly [3]. In patients with coronary fistulas, a coronary artery “steal” phenomenon can result in coronary blood preferentially passing through the fistula instead of the more distal myocardial capillaries. Angina has been reported in many patients with large fistulas and may be aggravated by distal coronary artery disease. In comparing CAF symptoms between adults and children, children more often have abnormal murmur and associated defects and rarely have CAF aneurysms and coronary artery disease. Symptoms are more likely to develop with advancing age, although treating asymptomatic CAF patients still poses management difficulties [4].

CAF are classified as distal or proximal. The proximal type arises near the origin of the coronary artery. A short proximal segment of the feeding coronary artery may be dilated, but the distal end of the original coronary artery is thin. The original coronary branches responsible for blood flow steal are hard to identify. The distal type of CAF originates near the distal end of a branch coronary artery. The feeding coronary artery proximal to a distal fistula gives rise to coronary branches that supply the myocardium [5]. This case was a right distal CAF, in which the left coronary branches responsible for blood flow steal could not be identified.

Therapies to close congenital CAF during childhood have been recommended to avoid complications such as myocardial ischemia, congestive heart failure, endocarditis, and aneurysmal dilatation [2]. Whether to treat CAF surgically or by percutaneous intervention in childhood is controversial. Of the acute complications following coil embolization, the most important are myocardial ischemia and conduction system disorders. Early complications have been reported to include transient ST-T wave changes, transient arrhythmias, distal coronary spasm, and fistula dissection [6]. In addition, there are many variations of the origin and course of coronary arteries in congenital heart disease, making percutaneous coronary intervention technically difficult. Asymptomatic children with large CAF who underwent therapeutic intervention have been reported [7]. Catheter techniques are difficult or impossible in a small percentage of patients, particularly younger pediatric cases, due to extreme vessel tortuosity and inability to deliver a catheter far enough distally [8]. The single coronary artery seen in this case is especially rare, making percutaneous coronary intervention technically difficult.

A balloon occlusion test before coil embolization of a CAF is necessary to avoid complications, particularly when many coronary branches cannot be distinguished clearly in small pediatric patients [9].

## 4. Conclusion

In patients with CAF, the coronary artery branches sometimes cannot be identified due to the presence of blood flow steal. In such cases, myocardial ischemia and conduction system disorders can be identified by performing the occlusion test before embolization.
